# La lipoatrophie annulaire des chevilles de découverte fortuite

**DOI:** 10.11604/pamj.2015.21.255.7485

**Published:** 2015-08-06

**Authors:** Samia Frioui, Faycel Khachnaoui

**Affiliations:** 1 Service de Médecine Physique et de Réadaptation Fonctionnelle, CHU Sahloul, Faculté de Médecine “Ibn El Jazzar”, Sousse, Tunisie

**Keywords:** Lipoatrophie annulaire des chevilles, Maladies auto-immunes, hyperthyroïdie, Annular lipoatrophy of the ankle, Autoimmune diseases, hyperthyroidism

## Image en medicine

La lipoatrophie annulaire des chevilles est une entité rare et méconnue. Seulement onze cas ont été rapportés dans la littérature. C'est une hypodermite lipoatrophiante localisée caractérisée par un état inflammatoire du tissu graisseux sous-cutané évoluant rapidement vers une lipoatrophie séquellaire. Elle touche surtout l'enfant et l'adulte jeune. Les différentes observations rapportées mettaient en évidence l'association fréquente de cette maladie à des dysthyroïdies. Son traitement est décevant. Cependant, de bons résultats ont été rapportés avec la corticothérapie générale et les antipaludéens de synthèse. Nous présentons le cas d'une patiente âgée de 54 ans aux ATCDS d'hypovitaminose D, d'hyperthyroïdie, d'arthralgies de type inflammatoire et de myalgies. Elle a été opérée à l’âge de dix ans pour genu varum bilatéral et il y a une année pour prothèse totale de la hanche gauche sur coxarthrose. Elle a été transférée dans notre service pour prise en charge rééducative post opératoire d'une prothèse totale de la hanche droite. L'examen montrait une femme de petite taille (150 cm) présentant un syndrome extrapyramidal. L'examen des membres trouvait des troubles trophiques des membres inférieurs avec au niveau du tiers inférieur des deux jambes remontant jusqu'aux mollets, une bande circonférentielle atrophique de onze centimètres de largeur touchant les chevilles. En reprenant l'interrogatoire nous avons constaté que la maladie avait débuté brutalement il y a cinq ans par des placards inflammatoires du tiers inférieurs des jambes qui ont évolué en quelques mois vers une lipoatrophie circonférentielle des chevilles. Devant l'ancienneté de l'atteinte nous avons opté pour l'abstention thérapeutique.

**Figure 1:**
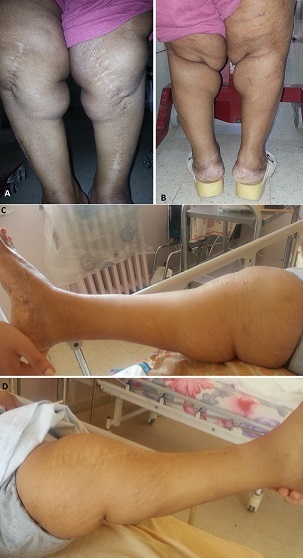
(A) vue de face: bande circonférentielle atrophique du tiers inférieur des deux jambes; (B) vue du dos: atrophie annulaire du tiers inférieur des deux jambes; (C) vue de profil: bande circonférentielle atrophique du tiers inférieur de la jambe droite; (D) vue de profil: bande circonférentielle atrophique du tiers inférieur de la jambe gauche

